# Effectiveness of a Newly Developed Instillation Aid for Unit-Dose Ophthalmic Solutions

**DOI:** 10.3390/jcm14155243

**Published:** 2025-07-24

**Authors:** Airi Takahashi, Yuka Kasai, Masako Sakamoto, Yuji Matsuda, Yuka Ito, Hirotaka Haro, Kenji Kashiwagi

**Affiliations:** 1 Department of Ophthalmology, Faculty of Medicine, University of Yamanashi, Yamanashi 409-3898, Japan; iairi@yamanahi.ac.jp (A.T.); kasayuka@yamanashi.ac.jp (Y.K.); masakok@yamanashi.ac.jp (M.S.); 2 Division of Rehabilitation, University of Yamanashi Hospital, Yamanashi 409-3898, Japan; ymatsuda@yamanashi.ac.jp (Y.M.); haro@yamanashi.ac.jp (H.H.); 3 Department of Nursing, University of Yamanashi Hospital, Yamanashi 409-3898, Japan; yukaf@yamanashi.ac.jp

**Keywords:** eye drop aid, unit-dose eye drops, glaucoma

## Abstract

**Background/Objectives:** To evaluate the effectiveness and limitations of a newly developed unit-dose eye drop instillation aid in patients with glaucoma. **Methods:** Hospitalized adult glaucoma patients at the University of Yamanashi were enrolled if they had self-administered glaucoma eye drops for at least six months, had no upper limb impairments or cognitive decline, and had corrected visual acuity of ≥20/200 in at least one eye. This study used 0.1% hyaluronic acid mini-ophthalmic drops. Eye drop instillation was performed in the following order: without aid in the sitting position, with aid in the sitting position, without aid in the supine position, and with aid in the supine position. One practice trial with the device was conducted beforehand. Successful instillation was defined as delivery of a drop into the conjunctival sac without contact with the ocular surface, eyelashes, or face. Patients were also surveyed regarding the perceived usefulness of the device. **Results:** Sixty-three patients (37 males, 26 females; mean age 71.3 ± 11.2 years) participated. In the sitting position, the success rate improved significantly from 70.3% without the aid to 89.1% with the aid (*p* = 0.0005). Success rates decreased with age but improved more markedly in older patients. In the supine position, the rate was 76.6% without the aid and 100% with the aid (*p* < 0.0001). **Conclusions:** Unit-dose eye drop aids significantly increase the success rate of instillation, especially among elderly patients, and may contribute to better adherence and treatment outcomes in glaucoma care.

## 1. Introduction

Glaucoma is a leading cause of visual impairment [1]. Aging is a significant risk factor for glaucoma, and, with an aging population, the number of elderly glaucoma patients is expected to increase in the future [1].

The most effective treatment for glaucoma is lowering intraocular pressure (IOP). Medical treatment using eye drops to reduce IOP plays a central role, and research has led to an increase in the variety of glaucoma eye drops available [2].

For these treatments to be effective, eye drops must be correctly applied to the conjunctival sac. Incorrect application or contact with surrounding areas can lead to side effects. To treat glaucoma accurately and safely, it is essential to use eye drops properly.

Various factors have been reported as causes of eye drop failure [3,4,5]. Davis et al. reported that 18.2–80% of patients contaminate the eye drop container by allowing it to contact their eyes or face, 11.3–60.6% administer excessive amounts of eye drops, and 6.8–37.3% miss their eyes and drop the medication elsewhere [6]. In our study, we investigated the causes of failure in eye drop administration among 103 glaucoma patients with a history of eye drop use for more than six months. The overall failure rate was 61.2%, and the contributing factors included advanced age, limited cervical spine extension angle, reduced hand pinch strength, worsening upper limb motor function disorders, ataxia, decreased best-corrected visual acuity, and visual field defects. The specific reasons for failure included administering drops outside the conjunctival sac (76.2%), contact between the tip of the eye drop bottle and other surfaces (22.2%), and administering more than two drops incorrectly (11.1%) [5].

Many commercial eye drops contain preservatives, but preservative components such as benzalkonium chloride (BAC) in these eye drops can cause adverse effects on the ocular surface [7,8,9,10,11,12]. The majority of eye drops use preservatives in their solutions to extend the shelf-life, convenience, and cost-effectiveness of their medications. Benzalkonium chloride (BAK) has been used in ophthalmology since the 1940s. It is by far the most common preservative and is found in more than half of all eye drops [13,14]. Many previous studies reported BAK-induced toxic effects on the ocular surface [15,16]. Many patients with chronic ocular diseases, such as glaucoma, use multiple types of eye drops, and preservatives in multidose eye drops are crucial for maintaining sterility but can be toxic to the ocular surface [12]. To reduce preservative-induced adverse effects, alternative preservative compounds, including polyquaternium-1, stabilized oxychloro-complexes, sodium perborate, and ionic-buffered preservatives, have been introduced to reduce preservative-associated adverse effects. In addition, eyedrop bottles with new dispensing mechanisms that allow longer-lasting preservative-free methods have also been introduced. However, these methods are not widely accepted.

To mitigate such side effects, preservative-free eye drops and single-dose unit (SDU) eye drops have become increasingly popular. Recently, there has been a trend toward unit-dose preparations of glaucoma drops to eliminate the need for preservatives [17]. Pisella et al. reported that, compared with preservative eye drops, preservative-free eye drops significantly reduce ocular surface symptoms and complications [18].

Although the handling of unit-dose pipettes is similar to that of conventional eye drop bottles [19], owing to their smaller bottle size than the refillable type, SDU eye drops can be difficult to handle, making them challenging for some patients to use [20]. While some assistive devices for SDU eye drops are available, they are often limited to specific products, presenting additional challenges [21].

In this study, we developed an assistive device specifically designed for SDU eye drops. Here, we report on the usefulness and challenges associated with this device.

## 2. Patients and Methods

This study was performed in accordance with the Declaration of Helsinki. All the participants provided written informed consent. The Ethics Committee of the University of Yamanashi School of Medicine (Approval Code 1574) approved this study.

**Figure 1:**
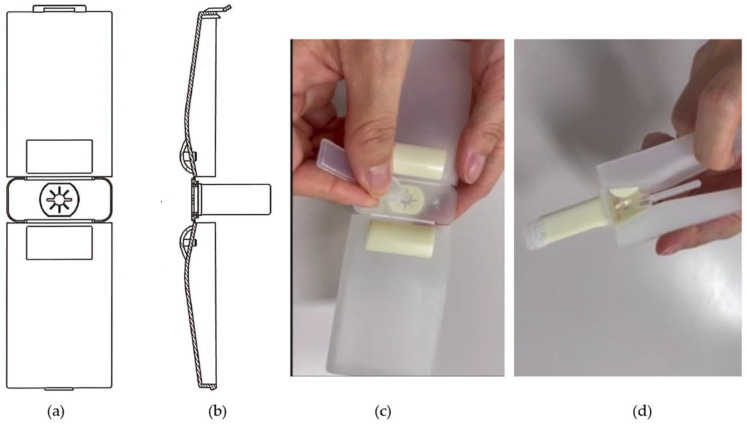
Eye drop aids: (**a**) top view, (**b**) side view, (**c**) attachment of the eye drop bottle, (**d**) attachment of the eye drop bottle.

**Eye drop aid (Figure 1)**

The demonstration video demonstrates the attachment of the SDU and the proper use of the eyedrop aid. In brief, this aid is foldable and can hold several bottles of SDU eye drops inside. During installation, the eye drop aid folds back and unfolds. The tip of the SDU is exposed by removing the lid, which is then attached to the aid via its ring-shaped holder. The aid contacts the face at two points: the eyebrow area and the upper cheek area. It includes a wing-shaped grasping section that allows a single drop of ophthalmic solution to be dropped with a single, normal operation. The entire eye drop aid is made of heat-resistant material that can be disassembled and sterilized by boiling. A feature of the aid is its ability to accommodate a lack of neck retroflexion because the angle of contact with the face is not vertical. Moreover, a single drop can be instilled with less force because white wings are inclined for eye drop application. Currently, all commercially available SDUs can be attached to this eye drop aid. This aid is not commercially available at the moment, but it is planned to be released at an affordable price for many patients. It is made from high-durability resin, making it highly resistant to damage under normal conditions of use.

**Patients**

This study was conducted from 1 September 2019 to 31 October 2020. Consecutive adult patients admitted to the University of Yamanashi Hospital for glaucoma treatment who met the following criteria were included: a history of self-administered glaucoma eye drop use for at least 6 months, no upper limb loss or motor impairment, no physical or cognitive decline that interferes with daily living, and a corrected visual acuity of 20/200 in at least one eye. Patients who did not meet these criteria or were deemed by the person in charge to be unable to complete this study for other reasons were excluded.

**Experimental design and definition of instillation failure**

A 0.1% hyaluronic acid ophthalmic solution (Santen Pharmaceutical Co., Ltd., Osaka, Japan) was used to assess the success of eye drop instillation. Each patient underwent eye drop instillation while in the sitting position. After the use of eyedrop aids was explained, they practiced instilling eye drops while they were in the sitting position. Patients then underwent eye drop instillation with an eye drop aid while they were in the sitting position. Next, the patients were administered eye drops in the supine position, first without the instillation aid and then with it. In each scenario, the eye drop test was performed twice, and success was judged on the second occasion. Two evaluators (Y.M. and Y.I.) assessed the success of eye drop instillation. Instillation failure was defined as one or more of the following conditions: the tip of the eye drop bottle directly touching the surface of the eye, eyelid, eyelashes, or face, or more than two drops of eye drops in a single trial.

**Investigated outcomes**

The following points were examined in this study: 1. the failure rate and factors associated with normal eye drops; 2. the eye drop success rates in the sitting and supine positions; and 3. the eye drop success rates in the sitting and supine positions, with and without the eyedrop aid. Patients were asked to complete a descriptive questionnaire about the ophthalmic aid.

**Routine ophthalmic examinations**

All patients underwent routine ophthalmic examinations, including best-corrected visual acuity, slit-lamp, and fundus examinations, within one month before entry. The corrected visual acuity was converted to logMAR (minimum angle of resolution) for the statistical analysis, and the best corrected visual acuity from both eyes was employed for the analysis. Visual field testing was performed via the Humphrey Visual Field test program 24-2 (Carl Zeiss Meditec, Inc., Dublin, CA, USA). The latest results of a visual field test performed within three months before entry were used. Better values of the mean deviation (MD) and upper and lower total deviation (TD) between the right and left eyes were subjected to statistical analysis.

**Questionnaire survey**

Patients were interviewed after this study to share their impressions of the eyedrop aid.

**Statistical analysis**

We compared the investigated factors between the instillation success group and the instillation failure group. A comparative study was conducted via the Mann–Whitney U test for continuous variables, Spearman’s signed-rank test for rank variables, and the chi–square test or Fisher’s test for categorical variables. *p* values less than 0.05 were considered significant. The results are presented as the means ± standard deviations.

## 3. Results

There were 63 patients (37 males and 26 females, mean age 71.3 ± 11.2 years) who met the inclusion and exclusion criteria and were enrolled in this study (Table 1). All the patients were right-handed, and daily eye drop application was performed while the participants were in a seated position with their right hand.

### 3.1. Success Rate of Eye Drop Instillation in the Sitting Position Without Eye Drop Aids

**Figure 2:**
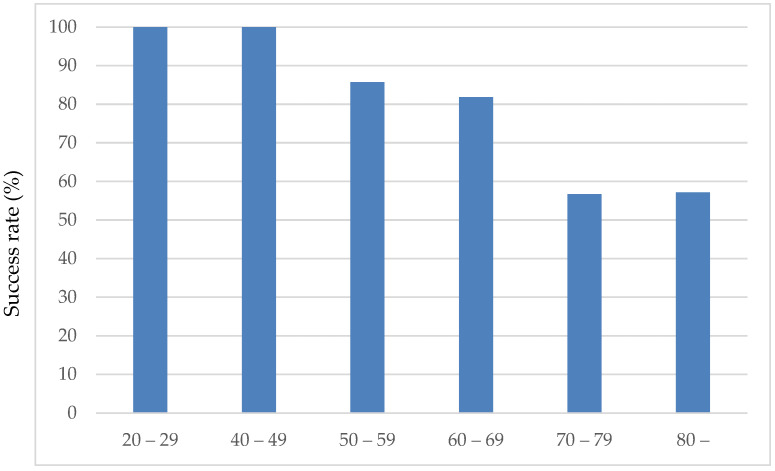
Success rate of eye drop instillation in the sitting position without eye drop aids.

The success rate of eye drop instillation in the sitting position without eye drop aids was 71.4% (45/63 patients). There was no significant difference by sex: 70.2% (11/37) for males and 73.01% (7/26) for females. The mean age of the successful group was 66.6 ± 10.8 years, and the mean age of the unsuccessful group was 78.0 ± 8.9 years, which was significantly greater than the mean age of the successful group, which was 66.6 ± 10.8 years (*p* = 0.001, Mann–Whitney U test). As shown in Figure 2, the eye drop instillation success rate decreased with age. Sixty percent (3/5) of the patients were in the 59-year-old and younger group, 89.5% (17/19) were in the 60-year-old group, 79.2% (19/24) were in the 70-year-old group, and 33.3% were in the 80-year-old and older group. The success rate of ophthalmic eye drops was lower in patients in their 80s and older, especially in those aged 80 years and older, at 33.3% (5/15). No significant effects of sex, best-corrected visual acuity, or MD values were observed on the success of eye drop instillation.

### 3.2. Success Rate of Eye Drop Instillation in the Sitting Position with the Use of an Eye Drop Aid

The success rate of eye drop use with the eye drop aid significantly improved to 90.5% (57/63 patients) (*p* = 0.0005). The reasons for unsuccessful eye drop placement were eye drops on the lower eyelid (four patients) and multiple factors (two patients).

### 3.3. Comparison of the Success Rates of Eye Drop Instillations in the Sitting and Supine Positions and the Effectiveness of the Eye Drop Aid (Figure 3)

The success rate of eye drop instillation in the supine position was 77.8% (49/63 patients) without an eye drop aid, which was significantly (*p* < 0.0001) better than that without an eye drop aid in the sitting position. The reasons for unsuccessful eye drop use were contact of the tip of the SDU with the eyelashes (seven patients), contact with the lower eyelid (four patients), and multiple reasons (three patients). The success rate was 100.0% when an eye drop aid was used.

### 3.4. Factors Associated with Improved Success Rates with the Use of Eye Drop Aids

In a study of factors associated with the transition from unsuccessful to successful eye drop instillation according to age, age was a significant variable in both the sitting (*p* = 0.003) and supine (*p* = 0.005) positions.

### 3.5. Results of the Subject Questionnaire

The participants reported several advantages, including ease of pressing the device and simple instillation of the drops. On the other hand, there are several issues, such as the following: it is difficult to remember how to use, it is difficult to determine the position to apply the aid, it is difficult to understand where the eye drops come out, and the device is too large.

## 4. Discussion

The present study assessed the efficacy of an SDU eye drop aid designed to facilitate the instillation of glaucoma eye drops. The findings demonstrated several important points. Despite being accustomed to glaucoma eye drop use, 28.6% of patients failed to instill the drops correctly. The success rate of eye drop instillation was significantly greater in the supine position than in the seated position. Advanced age was identified as a significant factor associated with instillation failure. The use of the aid significantly improved the instillation success rate, suggesting that it may be particularly advantageous for elderly patients.

Several previous studies have examined the success rate of eye drop instillation, with reported rates ranging from 18.2 to 80% [3,4,5,6]. In the present study, the success rate was 71.6%, which falls within the range reported in the literature. However, most previous studies utilized refill-type ophthalmic solutions, and few reports have focused on SDU-type solutions, such as those used in this study. In the literature, various factors contributing to unsuccessful eye drop instillation have been identified [3,4,5,6]. Among the most commonly cited factors are insufficient retroflexion of the head and advanced age. In our previous study, both the retroflexion angle and patient age were found to be associated with the success of eye drop instillation. Although the retroflexion angle was not evaluated in the present study, age was again found to be significantly associated with instillation success.

Patients with chronic ocular diseases, including glaucoma and dry eye, often require long-term topical treatment with eye drops. For some patients, the use of eye drops is challenging because of adverse effects caused by preservatives. These patients need to use either preservative-free SDU eye drops or specially designed bottles equipped with filters that prevent preservatives from reaching the ocular surface. However, filter-type eye drop bottles are relatively expensive and require considerable force for instillation. The use of SDU-type ophthalmic solutions has been expanding in recent years; however, the small size of the bottles often makes them difficult to handle. This challenge is particularly pronounced among elderly patients, who frequently experience declines in activities of daily living (ADLs), including reduced fingertip pinch strength. In our previous study, we demonstrated that impaired upper limb function and decreased fingertip pinch strength are contributing factors to unsuccessful eye drop instillation [5]. Furthermore, we found that the hardness of ophthalmic solution bottles varies by type among refill-type eye drops [22], with SDU-type bottles being harder and requiring more force to dispense a single drop than refill-type bottles. In contrast, the present ophthalmic aid device requires only a mild squeezing force, allowing a single drop to be dispensed with normal operation. These findings indicate a strong need for an SDU-type ophthalmic aid.

In the present study, the success rate of eye drop instillation was significantly greater in the supine position than in the seated position, a finding that is consistent with the report by Naito et al. [4]. Although adopting the supine position is one strategy to facilitate eye drop instillation, there are situations—particularly with the frequent use of SDU-type eye drops—where supine positioning is impractical. In such cases, the aid described here may offer a useful alternative. This study was conducted using hyaluronic acid unit-dose eye drops. Although preliminary investigations have confirmed that this aid can be used with other SDU eye drops, further studies are needed to validate its applicability to other eye drops.

In this study, all patients had experience self-administering eye drops, and each instillation trial was performed twice to ensure accuracy. Although instructions and practices were provided for the aid to minimize learning effects, some learning effects related to SDU eye drops may have remained. The present study did not assess whether the subjects had received prior training in eye drop instillation. Although the potential influence of this factor on the results cannot be entirely excluded, the enrolled patients reported no difficulties in using eye drops.

This study had several limitations. First, the number of participants was relatively small, and all patients had been receiving eye drop treatment for at least six months; thus, patients who were less accustomed to eye drop use were not evaluated. The patients included in this study did not have significant physical disabilities or severe visual impairments. In the future, it will be necessary to evaluate the effectiveness of this aid in patients with substantial physical disabilities or severe visual impairment, as these conditions are likely associated with unsuccessful eyedrop instillation. Second, only a single evaluation was performed without the use of supportive medications, which may have led to inaccurate assessments. Third, the study population was limited to patients with glaucoma, and individuals with other ocular diseases were not included.

This aid can be sterilized by boiling; however, if users do not perform the sterilization process correctly, there is a risk of infection.

In the case of SDU, there is a potential risk of microbial contamination [23,24]. Therefore, to prevent ocular surface infections when an instillation aid is used, it is essential to ensure proper sterilization of the device and manage it with careful attention to infection control. Therefore, proper instruction is essential prior to use. The patients enrolled in this study had been self-administering eye drops for more than six months and were therefore familiar with the instillation procedure. To ensure the accuracy of the success assessment, each instillation was performed twice. As the patients had no prior experience using the eye drop aid, they received instructions and were allowed to practice before the evaluation. Although these steps were intended to minimize the learning effect associated with the aid, the learning effect related to the SDU eye drops may not have been fully eliminated. Although the aid can be used with a variety of ophthalmic solutions, only hyaluronic acid eye drops were evaluated in this study, and other formulations were not examined. Additionally, in a patient survey, some participants noted that the aid was large and difficult to operate. Thus, further improvements to the device design are necessary.

## 5. Conclusions

A substantial proportion of patients, even those accustomed to glaucoma eye drop use, fail to instill them correctly. Proper eye drop instillation is fundamental for effective topical drug therapy. The SDU-type eye drop aid developed in the present study may serve as a useful tool to promote correct eye drop administration.

## 6. Patents

Kenji Kashiwagi holds a domestic patent in Japan related to the subject of this study.

## Figures and Tables

**Figure 3:**
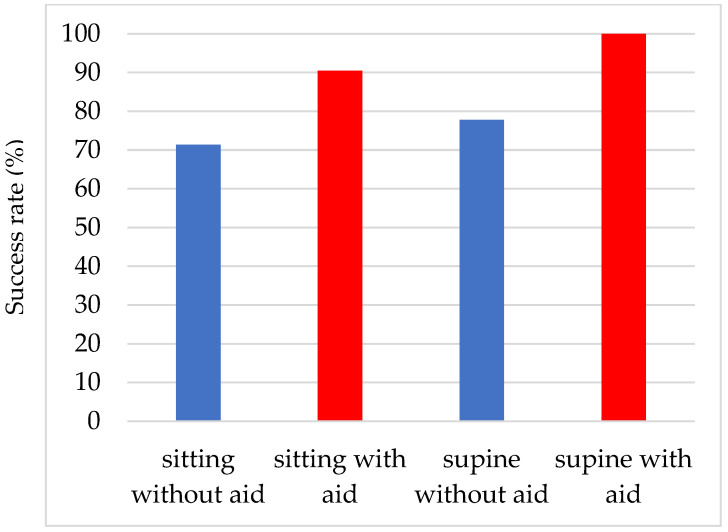
Comparison of the success rates of eye drop instillations in the sitting and supine positions and the effectiveness of the eye drop aid.

**Table 1:** Patient demographic characteristics.

Parameter	Value
patients (male:female)	63 (37:26)
Age (mean ± SD) (yrs)	71.3 ± 11.2
logMAR (mean ± SD)	Right: 0.282 ± 0.563 Left: 0.195 ± 0.284
HFA MD (dB)	Right: −15.1 ± 10.1 Left: −14.5 ± 9.6
Age group (yrs)	Number of patients (%)
20–29	1 (1.6)
30–39	0 (0.0)
40–49	2 (3.2)
50–59	2 (3.2)
60–69	19 (30.2))
70–79	24 (38.1)
80-	15 (23.8)

## Data Availability

All data supporting the findings of this study are available upon reasonable request.

## Abbreviations

The following abbreviations are used in this manuscript:

SDU	Single-dose unit
IOP	Intraocular pressure
